# Supplementary material for: GraphTyper2 enables population-scale genotyping of structural variation using pangenome graphs
Source: Nat Commun. 2019 Nov 27;10:5402. doi: 10.1038/s41467-019-13341-9 (PMC6881350; doi:10.1038/s41467-019-13341-9)
Supplement: Supplementary file 1 — Supplementary Information [file 41467_2019_13341_MOESM1_ESM.pdf]

Supplementary Information

"GraphTyper2 enables population-scale genotyping of structural variation using pangenome graphs"

Eggertsson et al.

Supplementary Figures

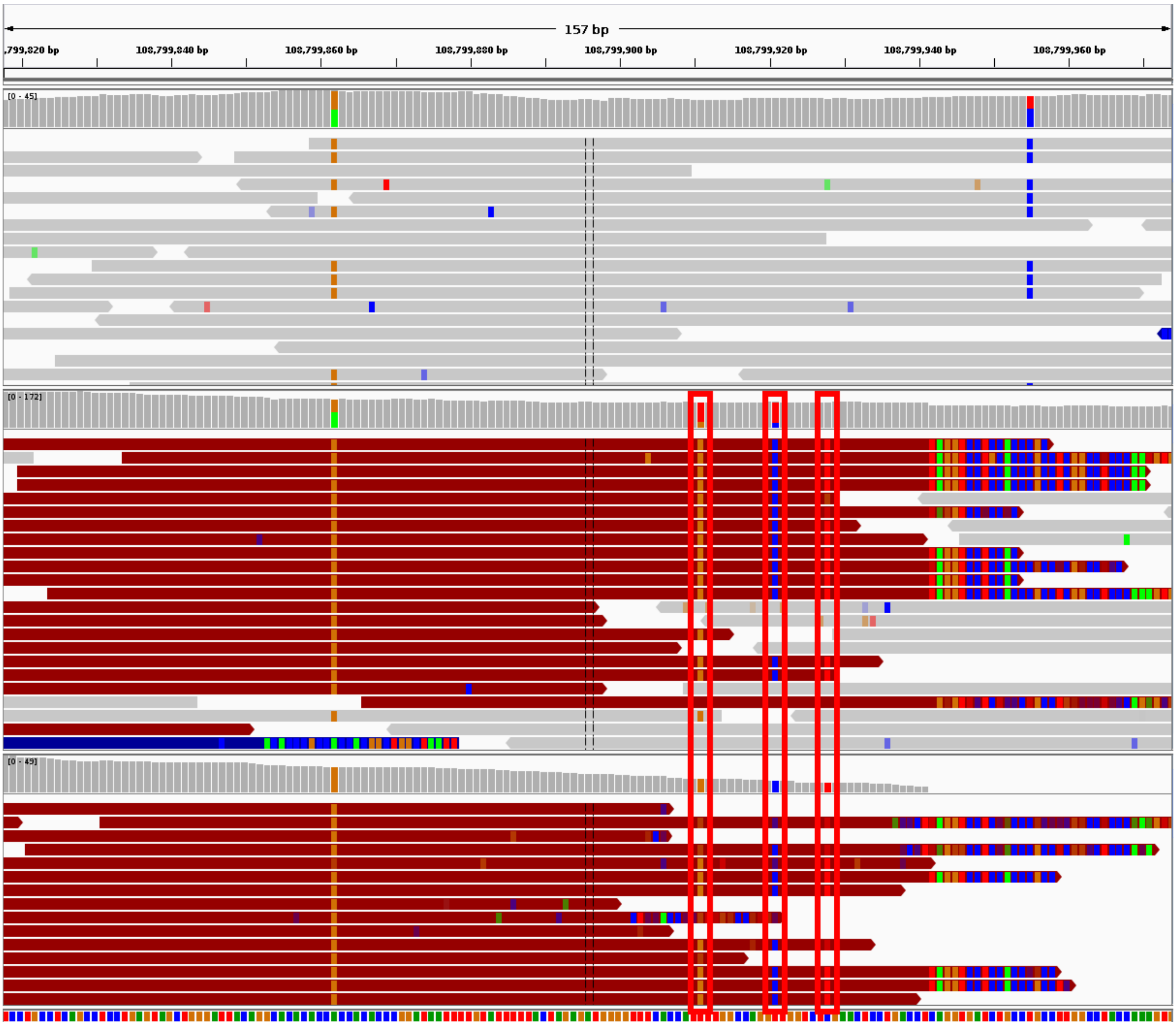

Supplementary Figure 1: Alignments of sequence reads of three Icelanders. A common 1163 bp deletion is at position chr4:108,799,896 (GRCh38). The top sample is not a carrier of the deletion, the sample in the middle is a heterozygous carrier, and the bottom sample is a homozygous carrier. The sequence reads overlapping the deletion breakpoint are mistakenly aligned into the deleted part of the reference such that three SNPs might be falsely genotyped due to misalignments (shown in red squares). The read alignments are visualized using IGV.

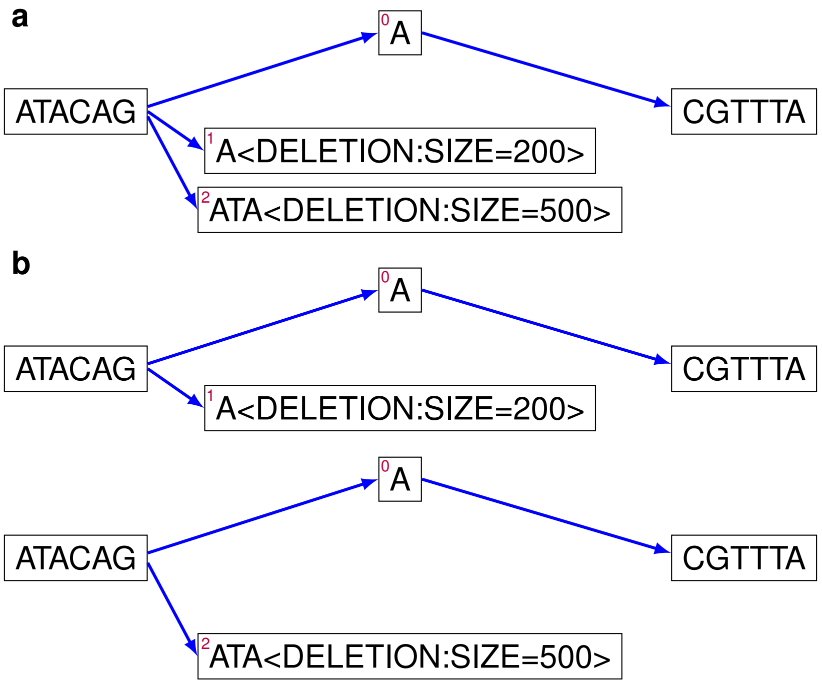

Supplementary Figure 2: GraphTyper graph with multi-allelic SV site. **a**. Graph with two SV deletion sites. The two SV alleles are tips in the graph where reads can align into the variant node, but not out of them since the variant node have no outgoing edges. **b**. Each alternative SV allele is compared against the reference allele separately, as if the sites were biallelic.

Supplementary Tables

Supplementary Table 1: High quality long-read assemblies used in the study.

		Autosomal SVs wrt.			
Sample ID	Population	hg19	GRCh38	Link to assembly data	Citation
AK1	Korean	14,616	14,827	`https://www.ncbi.nlm.nih.gov/Traces/wgs/?val=LPVO02`	[1]
CHM1	European	15,572	15,824	`https://www.ncbi.nlm.nih.gov/assembly/GCA_001297185.1`	[2, 3]
CHM13	European	15,142	15,419	`https://www.ncbi.nlm.nih.gov/assembly/GCA_000983455.2`	[4]
HX1	Han Chinese	11,403	11,590	`https://www.ncbi.nlm.nih.gov/assembly/GCA_001708065.2`	[5]
NA12878	Utah	23,579	23,725	`ftp://ftp.ebi.ac.uk/pub/databases/ena/wgs/public/oc/OCVW02.fasta.gz`	[6]
NA19240	Yoruba	13,351	13,541	`https://www.ncbi.nlm.nih.gov/assembly/GCA_001524155.1`	[7]

Supplementary Table 2: Variant transmission in parent-offspring trios. Percentages of an observed offspring genotype (columns) given the genotypes of its parents (rows). n is the number of observations in each row for the corresponding genotyping method.

	Manta+GraphTyper				Manta				smoove				Delly			
	n	0/0	0/1	1/1	n	0/0	0/1	1/1	n	0/0	0/1	1/1	n	0/0	0/1	1/1
0/0+0/0	3044	99.8%	0.2%	0.0%	31546	97.1%	2.9%	0.0%	5586	92.3%	7.4%	0.3%	69994	91.2%	8.8%	0.0%
0/0+0/1	1500	50.3%	49.6%	0.1%	6096	74.4%	25.2%	0.4%	2701	54.6%	44.2%	1.1%	31652	52.9%	46.6%	0.5%
0/0+1/1	179	2.2%	97.2%	0.6%	363	42.4%	51.8%	5.8%	313	33.2%	50.2%	16.6%	2229	2.1%	92.4%	5.5%
0/1+0/1	913	18.3%	63.3%	18.4%	3058	34.2%	62.6%	3.2%	3514	17.5%	71.5%	11.0%	66962	12.0%	84.6%	3.4%
0/1+1/1	689	0.6%	47.8%	51.7%	388	13.7%	41.5%	44.8%	1170	7.9%	42.1%	50.0%	9889	0.7%	51.3%	48.0%
1/1+1/1	1118	0.0%	0.4%	99.6%	292	12.0%	0.3%	87.7%	2139	6.4%	5.2%	88.4%	9064	0.0%	2.1%	97.9%

Supplementary Table 3: Number of validated SVs in Icelanders using Manta+GraphTyper. An SV is considered validated if it passed all filters and has at least two supporting long-reads.

	Validated SVs			Total SVs		
Genome	Deletions	Insertions	Total	Deletions	Insertions	Total
1	3378	3166	6544	5252	5401	10653
2	3627	3544	7171	5577	5441	11018
3	3694	2859	6553	6570	4429	10999
4	3601	3426	7027	5433	5335	10768
5	3183	2990	6173	5242	5415	10657
6	3642	3253	6895	5857	5261	11118
7	3429	3189	6618	5488	5298	10786
8	3803	3482	7285	5612	5320	10932
9	3499	3367	6866	5537	5636	11173
10	3428	2771	6199	6380	4635	11015
11	3644	3405	7049	5302	5377	10679
12	3538	3363	6901	5673	5321	10994
13	3586	2869	6455	6525	4528	11053
14	3613	3575	7188	5189	5536	10725
15	3578	3278	6856	5596	5368	10964
16	3336	2816	6152	5852	5164	11016
17	3575	3372	6947	5986	5390	11376
18	3439	3134	6573	6077	5415	11492
19	3760	3631	7391	5279	5385	10664
20	3565	3516	7081	5190	5516	10706
21	3626	3499	7125	5509	5254	10763
22	3464	3235	6699	5503	5371	10874
23	3425	3220	6645	5541	5398	10939
24	3842	2981	6823	6482	4471	10953
25	3501	3385	6886	5293	5423	10716
26	3583	3561	7144	5266	5435	10701
27	3495	3287	6782	5226	5474	10700
28	3496	2749	6245	6339	4569	10908
29	3665	3576	7241	5216	5497	10713
30	3634	3256	6890	5776	5169	10945
31	3798	3573	7371	6072	5489	11561
32	3518	3416	6934	5299	5522	10821
33	3580	3369	6949	5263	5404	10667
34	3596	2978	6574	6287	4653	10940
35	3670	3464	7134	5826	5246	11072
36	3582	3237	6819	5584	5267	10851
37	3695	2970	6665	6488	4561	11049
38	3545	3326	6871	5928	5370	11298
39	3800	2998	6798	6527	4568	11095
40	3632	2857	6489	6404	4645	11049
41	3376	2901	6277	5925	5113	11038
Average	3571.8	3240.1	6811.8	5740.8	5196.9	10937.6

Supplementary Methods

Contents

1 Original GraphTyper publication 8

2 New features in GraphTyper since its original publication 13

3 High-confidence SV filter 15

4 Genotyping public data of parent-offspring trio 17

5 Experimental setups 20

6 Evaluations 24

7 External tools and dependencies 26

1 Original GraphTyper publication

The current publication is an extension of GraphTyper [8], we review its main data structures below.

GraphTyper's main data structure is a directed acyclic graph that encodes a reference pangenome graph, an extension of the traditional linear reference genome (Supplementary Figure 3a). In an idealized pangenome graph there exists a path that contains the genome of every sequenced individual. The data structure incorporates known variation to improve sequence alignments and may be able to align unaligned and clipped sequences. In GraphTyper combined sequence alignment and variant calling in a single step, which makes the method scale well with the number of samples. For example when genotyping 15,220 whole-genome samples we measured GraphTyper to take 60% less time than GATK Unified Genotyper [9] on the same samples.

GraphTyper performs variant calling in several iterations (Supplementary Figure 3b). During each iteration, GraphTyper updates the pangenome graph structure, and makes final variant calls in the last iteration. In the first iteration the reference sequence is given as input and, optionally, a set of known variants (Supplementary Figure 4a). The sequence reads are aligned to the graph and the variation within the graph is genotyped for each sample individually. Optionally, GraphTyper discovers novel SNPs and indels based on the read alignments and adds them to the graph structure in the following iteration.

The graph data structure consists of nodes, which are connected by directed edges. Sequences are associated with the nodes and the edges describe how a possible haplotype could be reconstructed (Supplementary Figure 4b). The index data structures contain

a key-value store where k-mers are keys and a list of their start and end positions are values, along with any variants they might overlap (Supplementary Figure 4c). In the figure we demonstrate how the algorithm works using $k = 5$, however we use $k = 32$ in GraphTyper.

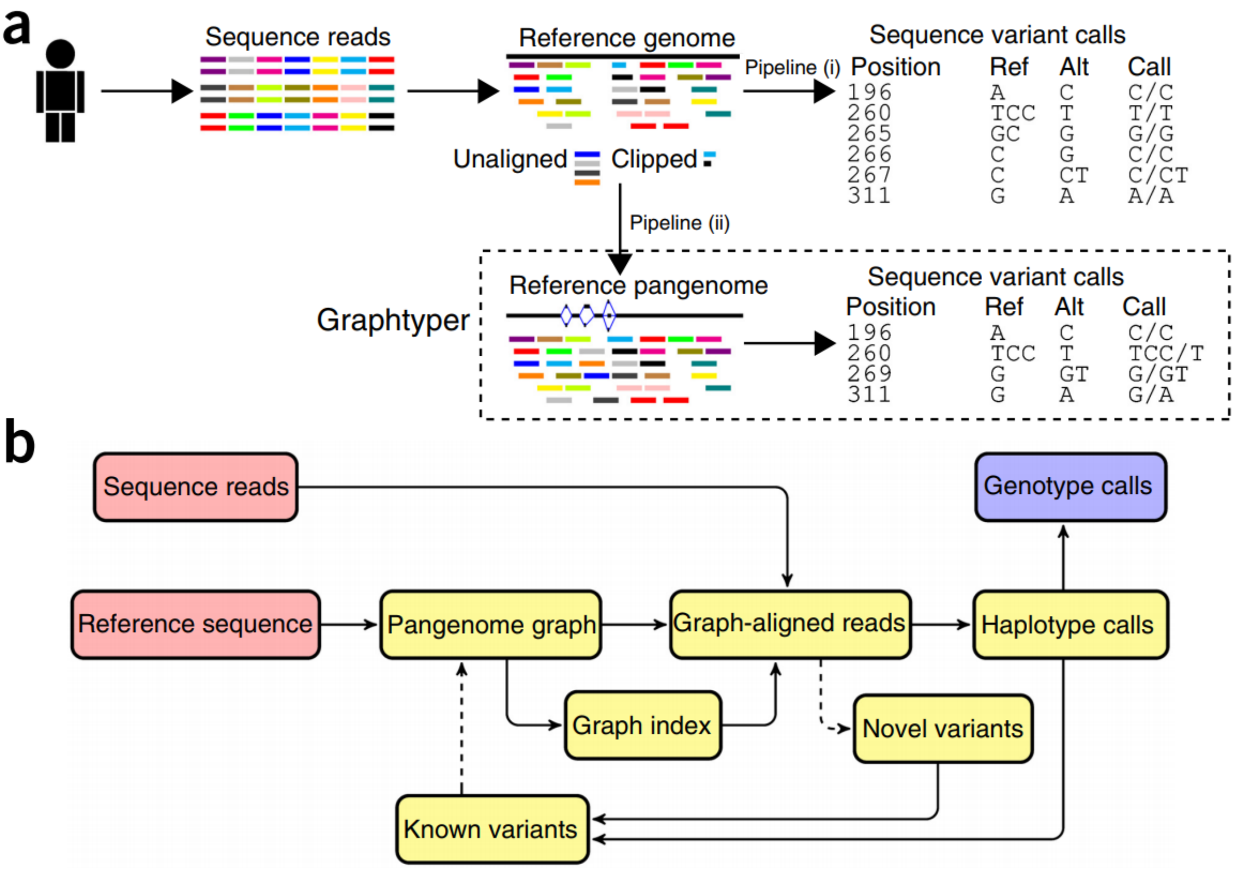

Supplementary Figure 3: Genotyping pipeline designs. **a**. Overview of two genotyping pipeline designs. Pipeline (i): a commonly used genotyping pipeline, where sequence reads are aligned to a reference genome sequence and sequence variants are called from discordances between the reads and the reference. Pipeline (ii): Graphtyper's genotyping pipeline. Sequence reads are realigned to a variants-aware pangenome graph and variants are called on the basis of which path the reads align to. **b**. Graphtyper's iterative genotyping process. Dashed paths are optional. As input, Graphtyper requires a reference genome sequence and sequence reads (red) and outputs genotype calls (blue) of variants.

a Reference sequence: ACCTCCAGACGTTTAGGGACCCCATTGAGTG

Known variants

Position	Reference	Alternative
9	A	C
19	A	ACC
27	GA	A
28	A	T

→

Known variants after merge

Position	Reference	Alternative
9	A	C
19	A	ACC
27	GA	GT,A

b

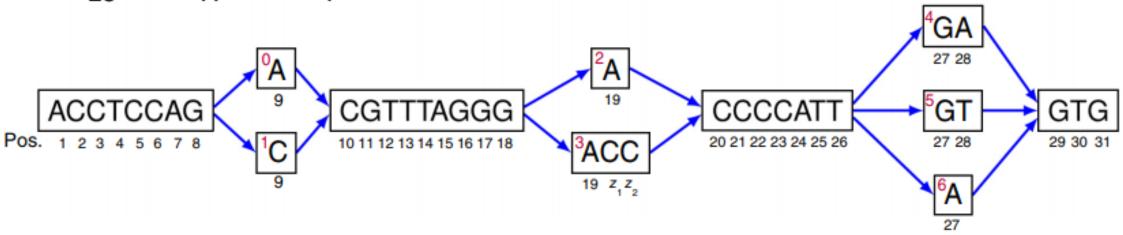

c

5-mer	Start pos.	End pos.	Variant ID	Start pos.	End pos.	Variant ID
ACCCC	19	23	2	19	21	3
ACCTC	1	5	NA			
ACGTT	9	13	0			
AGACG	7	11	0			
AGCCG	7	11	1			
AGGGA	15	19	2	15	19	3
...						
CCCAT	21	25	NA			
CCCCA	20	24	NA			
CCCCC	z_1	22	3	z_2	23	3
...						
GGACC	17	z_2	3	17	21	2
GGGAC	16	z_1	3	16	20	2
...						
TTGTG	25	29	5			
...						

d

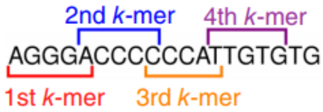

e

5-mer	Start pos.	End pos.	Variant ID	Start pos.	End pos.	Variant ID
AGGGA	15	19	2	15	19	3
ACCCC	19	23	2	19	21	3
CCCAT	21	25	NA			
TTGTG	25	29	5			

f

AGGGA ACCCC CCCAT TTGTG

AGGGC ACCCA CCCAA TTGTA
AGGGG ACCCG CCCAC TTGTC
AGGGT ACCCT CCCAG TTGTT
AGGAA ACCAC CCCCT TTGAG
AGGCA ACCGC CCCGT TTGCG
AGGTA ACCTC CCCTT TTGGG
AGAGA ACACC CCAAT TTATG
AGCGA ACGCC CCGAT TTCTG
AGTGA ACTCC CCTAT TTTTG
AAGGA AACCC CACAT TAGTG
ACGGA AGCCC CGCAT TCGTG
ATGGA ATCCC CTCAT TGGTG
CGGGA CCCCC ACCAT ATGTG
GGGGA GCCCC GCCAT CTGTG
TGGGA TCCCC TCCAT GTGTG

g

Seed	Start pos.	End pos.	Variant ID
AGGGACCCCCCATTGTG	15	29	3,5
AGGGACCCC	15	23	2

h

Extended longest seed	Start pos.	End pos.	Variant ID
AGGGACCCCCCATTGTGTG	15	31	3,5

Supplementary Figure 4: Graphtyper's graph and index data structures and sequence alignment algorithm. **a**. An example reference sequence and its known variation. All overlapping variants are merged. **b** Constructed pangenome reference graph. We draw the path of the reference sequence as the topmost path. **c**. The index data structure with $k = 5$. 5-mers in the graph are mapped to a list of its start position, end position, and a variant ID that it overlaps, if any. **d**. Four k-mers are extracted from a sequence read. Each k-mer overlaps its neighbor k-mer by one character. **e**. An example lookup of the k-mers from the index data structure from c. **f**. All extracted k-mers with a single substitution. **g**. Seeds are generated from matches in the index lookup. **h**. Final graph alignment after extending the longest seed.

2 New features in GraphTyper since its original publication

SV genotyping The main paper describes the addition of SV genotyping in GraphTyper. A few tweaks have been made to the base GraphTyper code to improve the quality when genotyping SVs. Most notably, we have changed the default parameters for read alignments to be considered when genotyping. We now only allow reads to have at most 3% error rate compared to the graph (previously it was 5%), we do not allow reads to a clipped graph alignment at either end, and we do not consider any reads shorter than 90 bp (which may happen if the reads have been shortened by removing adapters).

We have also changed the alignment algorithm slightly, such that if a read partially overlaps an allele, we only count it as supporting if there are at least 5 bp overlapping the allele. Reads that fail on this criterion are considered ambiguous, i.e. they have no effect on the called genotyped using the breakpoint model but may still used in the coverage model.

Other features In addition to enabling population-scale SV genotyping, we have added several new features to GraphTyper since its original publication. The major improvements are noted below.

We have added a new subcommand in GraphTyper called `discover` that will discover variants directly from read alignments of the global read aligner, i.e. BWA-MEM [10]. The subcommand is useful in the very first GraphTyper iteration when the pangenome graph contains only the reference haplotype and thus a graph realignment is not expected to improve the alignment. Since no realignment to a graph is done, the operation is much faster compared to discovering variants with the `call` command. We have also measured

that using `discover` subcommand results in similar or better recall so using instead of the `call` subcommand in the first iteration is highly recommended. We have updated our recommended GraphTyper pipelines (`https://github.com/DecodeGenetics/graphtyper-pipelines`) with the new subcommand.

We have also updated GraphTyper's variant discovery filters to reduce systematic false positive calls. In addition to checking for the number and fraction of reads supporting an alternative allele at a site, we require that for each alternative allele: (1) There must be some support from both read strands (forward and reverse). (2) At least one of the supporting reads must have a base-pair quality of 25 or more in the base-pairs that overlap the alternative allele. (3) There must be at least 2 unique read positions that overlap the variant. (4) There must be support by both first-in-pair reads and second-in-pair reads. (5) There must be at least 3 supporting reads that have a mate that maps to the same graph. We measured that these new criteria removed approximately 67% of non-germline calls (mostly false positive) in our population-scale genotyping, while having almost no effect (<0.1%) on germline recall.

We have also added support for working with any reference genome in GraphTyper. Originally GraphTyper had some hard-coded values that only worked for either the hg19 or the GRCh38 human references, but now we gather these values at graph construction and store them along with the graph. With the new update, contigs are accepted with any name and length. This features makes it possible to use GraphTyper for variant calling on other species than human, as long it has a reference genome available.

3 High-confidence SV filter

We used the following filters on the set of aggregated SVs in the Manta+GraphTyper dataset. For other datasets we used all variant that are flagged "PASS" in the FILTER field of the VCF file. We applied the filters using vcffilter from vcffib (`https://github.com/vcflib/vcflib`).

Breakends filter:

```
QD > 20 & ( ABHet > 0.30 | ABHet < 0 ) & ( AC / NUM_MERGED_SVS ) < 10 &
    PASS_AC > 0 & PASS_ratio > 0.1
```

Deletions filter:

```
QD > 12 & ( ABHet > 0.30 | ABHet < 0 ) & ( AC / NUM_MERGED_SVS ) < 25 &
    PASS_AC > 0 & PASS_ratio > 0.1
```

Duplications filter:

```
QD > 5 & PASS_AC > 0 & ( AC / NUM_MERGED_SVS ) < 25
```

Insertions filter:

```
PASS_AC > 0 & ( AC / NUM_MERGED_SVS ) < 25 & PASS_ratio > 0.1 & ( ABHet > 0.25
     | ABHet < 0 ) & MaxAAS > 4
```

Inversions filter:

```
PASS_AC > 0 & ( AC / NUM_MERGED_SVS ) < 25 & PASS_ratio > 0.1 & ( ABHet > 0.25
     | ABHet < 0 ) & MaxAAS > 4
```

Command The full command used to filter the VCF containing the aggregated calls was:

```
vcffilter -f "( SVTYPE = BND & QD > 20 & ( ABHet > 0.30 | ABHet < 0 ) & ( AC /
    NUM_MERGED_SVS ) < 10 & PASS_AC > 0 & PASS_ratio > 0.1 ) | ( SVTYPE = DEL
    & QD > 12 & ( ABHet > 0.30 | ABHet < 0 ) & ( AC / NUM_MERGED_SVS ) < 25 &
    PASS_AC > 0 & PASS_ratio > 0.1 ) | ( SVTYPE = DUP & QD > 5 & PASS_AC > 0
   & ( AC / NUM_MERGED_SVS ) < 25 ) | ( SVTYPE = INS & PASS_AC > 0 & ( AC /
   NUM_MERGED_SVS ) < 25 & PASS_ratio > 0.1 & ( ABHet > 0.25 | ABHet < 0 ) &
   MaxAAS > 4 ) | ( SVTYPE = INV & PASS_AC > 0 & ( AC / NUM_MERGED_SVS ) < 25
    & PASS_ratio > 0.1 & ( ABHet > 0.25 | ABHet < 0 ) & MaxAAS > 4 )" $(
   SV_AGGREGATED_VCF)
```

Filtering SV genotype calls In addition to filtering SV sites, we also created a filter that removes low quality SV genotype calls on a per sample basis at high-confidence SV sites. Delly, Manta and Manta+GraphTyper genotype calls were filtered using the FT (filter) field of the VCF: `vcffilter -g "FT = PASS" $(VCF)`. GraphTyper uses the following criteria on each sample call to determine if a call passes:

- All genotyping models must have at least 10 unique reads (reads that do not support more than one allele).
- If all genotyping models agree on the genotype, the lowest GQ in all models must be above 10.
- If the genotyping models do not agree which genotype to call, the highest GQ must be above 40 and no genotyping model can have a PHRED value above 20 for that genotype.

All criteria must pass such that the genotype call is passed in GraphTyper's filter.

4 Genotyping public data of parent-offspring trio

We evaluated the genotyping performance of GraphTyper on a well-studied parent-offspring trio (NA12878, NA12891, and NA12892). Whole-genome sequence data of these samples are publicly available from the Platinum Genome project [11]. The data is 101-bp paired-end Illumina HiSeq 2000 reads sequenced to 50x average coverage depth. We aligned the sequence reads to the human reference genome (hg19) using BWA-MEM [10]. We ran Manta [12] on all three samples independently and then merged all SV sites using svimmer. We also ran GraphTyper's small variant pipeline to construct a graph with SNPs and indels and added the SV sites discovered by Manta to the graph and genotyped the entire set of variants.

We compared the results from both of GraphTyper's deletion genotyping models to 2,612 high-confidence deletions found in the autosomes of NA12878, originally discovered using multiple sequencing technologies using svclassify [13]. We would expect to find fewer deletions with only short-read data. The high-confidence deletion set contains only begin and end coordinates of the deletions but no genotype information. We therefore only tested if the deletion allele was called (genotype 0/1 or 1/1) in the query sets. We allowed an offset of the breakpoint locations as they are not always accurately reported by the SV discovery tools.

The truth set contains only a small fraction of the expected number of deletions in a genome and is likely missing deletions that are harder to discover, e.g. deletions in repetitive regions. The number of deletions we consider true is therefore an under-estimate. However, we believe the truth set serves a purpose in comparing different genotyping

methods. We required both begin and end positions to be within the selected offset for a deletion to be considered recalled. Rather than arbitrarily selecting one offset threshold we compared the methods using different thresholds. We also subsampled the reads using samtools [14] and repeated the experiment using 3x and 10x average coverage.

Our results showed that the breakpoint model had higher sensitivity than the coverage model when coverage is 10x or more (Supplementary Figure 5a). As expected, the aggregated model had the highest sensitivity at all coverages with more than 90% of the deletions in the truth set recalled. In what follows, all GraphTyper evaluations were performed only on the aggregated model.

We also performed SV genotyping on the same parent-offspring trio using widely-used methods: Manta [12], Delly [15], BayesTyper [16], and smoove (Lumpy [17] (discovery) with SVTyper [18] (genotyping)) using appropriate filters. In our experiment, Manta+GraphTyper had the highest SV deletion sensitivity at all tested breakpoint precision thresholds (67.8%-90.7%) (Supplementary Figure 5b). BayesTyper had the highest precision when testing with a strict breakpoint precision threshold but Manta+GraphTyper had only a slightly lower precision (Supplementary Figure 5c). smoove had the highest precision when allowing a more lenient threshold. We also calculated the F1-score for each method (Supplementary Figure 5d) which ranks Manta+GraphTyper highest at lower breakpoint precision thresholds. Based on the above observations, we concluded that Manta+Graphtyper is a sensitive method to detect SVs.

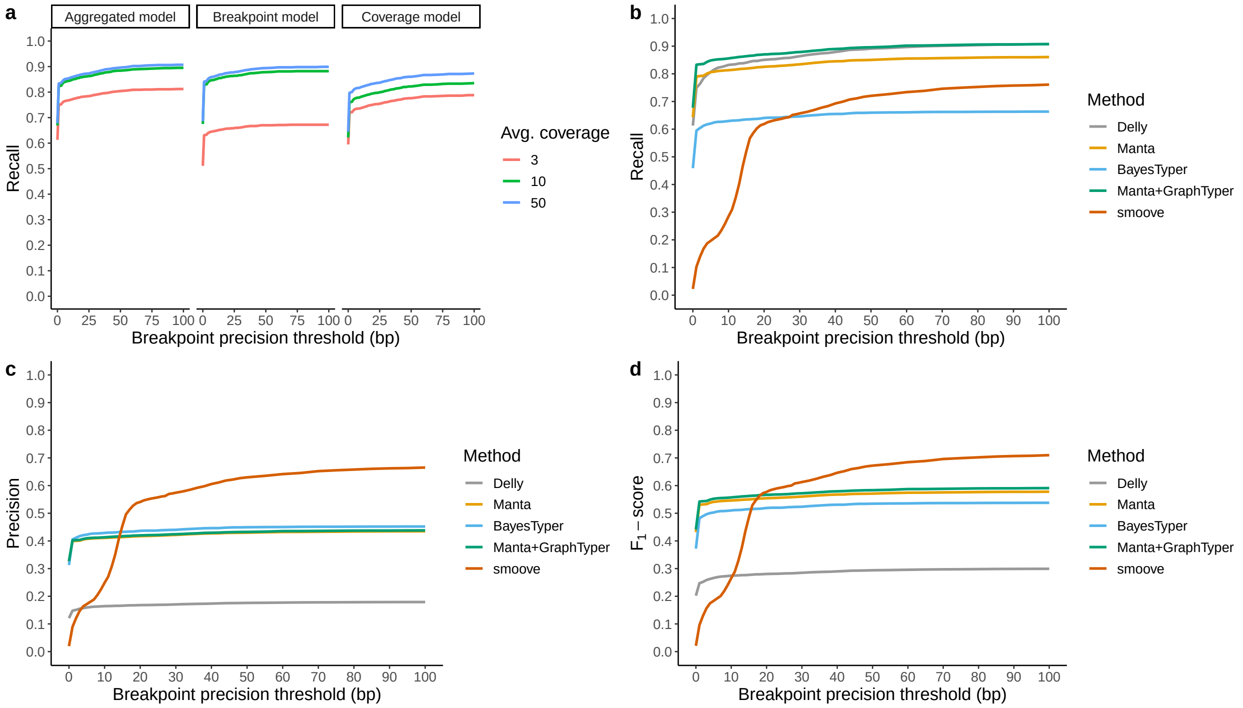

Supplementary Figure 5: Comparisons to svclassify's deletion truth set. The breakpoint precision threshold is the maximum number of allowed difference of begin and end positions between the query and svclassify's high-confidence set. **a**. Deletion sensitivity of GraphTyper's genotyping models in the Manta+GraphTyper pipeline. Comparison of each SV deletion genotyping model was compared against the svclassify high-confidence deletion set for NA12878. Sequence reads were subsampled to test how coverage affected sensitivity of each genotyping model. **b**. Deletion sensitivity comparison between Delly, Manta, BayesTyper, Manta+GraphTyper, and smoove using svclassify's high-confidence deletion set for NA12878 as a truth set. **c**. Deletion precision comparison. **d**. Deletion F1-scores comparison.

5 Experimental setups

In our experiments we used GraphTyper version 2.0, Manta version 1.4, Delly version 0.7.8, BayesTyper version 1.3.1, lumpy version 0.2.13 with SVTyper version 0.7.0, and smoove version 0.2.3. All experiments were run on deCODE's computer cluster.

We evaluated the set of SVs that these tools set as "PASS" in the VCF filter field, if available. The exact commands we used are shown below.

Manta + GraphTyper (syndip)

```
bin/configManta.py --referenceFasta=$(GENOME) --runDir CHM1_CHM13 --bam $(BAM)
    --referenceFasta=$(GENOME)
CHM1_CHM13/runWorkflow.py --mode=local --memGb=110 --jobs=24
```

We then used the GraphTyper SV pipeline (`https://github.com/DecodeGenetics/graphtyper-pipelines`) with `CHM1_CHM13/results/variants/diploidSV.vcf.gz` as the `SV_VCF` in the config.

Manta + GraphTyper (parent-offspring trio)

```
bin/configManta.py --referenceFasta=$(GENOME) --runDir NA12878 --bam NA12878.
   bam
bin/configManta.py --referenceFasta=$(GENOME) --runDir NA12891 --bam NA12891.
   bam
bin/configManta.py --referenceFasta=$(GENOME) --runDir NA12892 --bam NA12892.
   bam
NA12878/runWorkflow.py --mode=local --memGb=110 --jobs=24
```

```
NA12891/runWorkflow.py --mode=local --memGb=110 --jobs=24
NA12892/runWorkflow.py --mode=local --memGb=110 --jobs=24
ls NA128*/results/variants/diploidSV.vcf.gz > input_vcfs
svimmer input_vcfs `seq 1 22` | bgzip -c > merged.vcf.gz
tabix merged.vcf.gz
```

We then used the GraphTyper SV pipeline with `merged.vcf.gz` as the `SV_VCF` in the config.

Delly (syndip)

```
delly_v0.7.8_parallel_linux_x86_64bit call --genome=$(GENOME) --outfile=
    CHM1_CHM13.bcf --exclude human.hg19.excl.tsv $(BAM)
```

Delly (parent-offspring trio)

```
delly_v0.7.8_parallel_linux_x86_64bit call --genome=$(GENOME) --outfile=1_78.
    bcf --exclude human.hg19.excl.tsv NA12878.bam
delly_v0.7.8_parallel_linux_x86_64bit call --genome=$(GENOME) --outfile=1_91.
    bcf --exclude human.hg19.excl.tsv NA12891.bam
delly_v0.7.8_parallel_linux_x86_64bit call --genome=$(GENOME) --outfile=1_92.
    bcf --exclude human.hg19.excl.tsv NA12892.bam
delly_v0.7.8_parallel_linux_x86_64bit merge -o sites.bcf 1_78.bcf 1_91.bcf 1
    _92.bcf
delly_v0.7.8_parallel_linux_x86_64bit call --vcffile=sites.bcf --genome=$(
    GENOME) --outfile=2_78.bcf --exclude human.hg19.excl.tsv NA12878.bam
delly_v0.7.8_parallel_linux_x86_64bit call --vcffile=sites.bcf --genome=$(
    GENOME) --outfile=2_91.bcf --exclude human.hg19.excl.tsv NA12891.bam
delly_v0.7.8_parallel_linux_x86_64bit call --vcffile=sites.bcf --genome=$(
```

```
    GENOME) --outfile=2_92.bcf --exclude human.hg19.excl.tsv NA12892.bam
bcftools merge -m id -O z -o final_results.vcf.gz 2_78.bcf 2_91.bcf 2_92.bcf
tabix final_results.vcf.gz
```

Delly was run similarly for the 56 Icelandic samples (four large families).

Manta (syndip)

```
bin/configManta.py --referenceFasta=$(GENOME) --runDir=CHM1_CHM13 --bam=$(BAM)
CHM1_CHM13/runWorkflow.py --mode=local --memGb=110 --jobs=24
```

Manta (parent-offspring trio)

```
bin/configManta.py --referenceFasta=$(GENOME) --runDir=joint --bam=NA12878.bam
    --bam=NA12891.bam --bam=NA12892.bam
joint/runWorkflow.py --mode=local --memGb=110 --jobs=24
```

BayesTyper

We used the BayesTyper Snakemake workflow. The workflow was configured with Genome Analysis ToolKit version 3.6, Platypus (commit: cbbd9146183a2aba5f4884df36fbd58988133150), Manta version 1.4.0, bcftools 1.5 and KMC version 3.1.0.

smoove (syndip)

```
smoove call -x --name CHM1_CHM13 --fasta $(GENOME) -p 2 --genotype $(BAM)
```

smoove (parent-offspring trio)

```
smoove call -x --name svclassify --fasta $(GENOME) -p 2 --genotype NA12878.bam
    NA12891.bam NA12892.bam
```

smoove was run similarly for the 56 Icelandic samples (four large families).

Sniffles

```
sniffles --report_seq --ignore_sd -l 30 -d 1000 -s 2 -m $(INPUT_BAM) -v $(
   OUTPUT_VCF) -t 24 --num_reads_report 30 --genotype
```

We then post-filtered variants that were smaller than 50 bp and SVs that had breakpoints of different chromosomes.

6 Evaluations

Checking overlap with external SV datasets We also used svimmer for measuring the overlap of the external SV dataset to our callset. The 1000G SVs were obtained from: `http://ftp.1000genomes.ebi.ac.uk/vol1/ftp/phase3/integrated_sv_map/supporting/GRCh38_positions/ALL.wgs.mergedSV.v8.20130502.svs.genotypes.GRCh38.vcf.gz`. The file contains sites after their positions had been lifted over to GRCh38. The GoNL SVs were obtained from: `https://molgenis26.target.rug.nl/downloads/gonl_public/variants/release6.1/20161013_GoNL_AF_genotyped_SVs.vcf.gz` and lifted over from build 37 to 38. The Abel *et al.* SVs were obtained from the following zip file: `https://www.biorxiv.org/content/biorxiv/early/2018/12/31/508515/DC1/embed/media-1.zip?download=true`.

The following command was used to check overlap between our SV dataset and the external datasets:

```
svimmer <(echo $(EXTERNAL_VCF); echo $(GraphTyper_VCF)) --max_distance $(
  DISTANCE) --max_size_difference -1 --ignore-types --join-mode chr{1..22} |
   bgzip -c > $(OUTPUT)
tabix $(OUTPUT)
```

Variants that have `NUM_JOINED_SVS` greater than 1 are SVs in external dataset but are also found in Iceland. The following values for `$(DISTANCE)` were tested in our analysis: 1, 3, 5, 7, 9, 11, 13, 15, 17, 20, 23, 26, 30, 35, 40, 50, 70, and 100. We needed to use the --ignore-types option since there were many inconsistencies in the classifications of SV types between datasets. For example, in the 1000G SV set there are many deletions/duplications classified as "CNV" while the other datasets separate these. When filtering

based on allele frequency we used the `EUR_AF` in the 1000G dataset and AF in the other datasets.

Long-read validation We also used svimmer for joining GraphTyper SVs with Sniffles SVs in our long-read validation experiment.

```
svimmer <(echo $(GraphTyper_VCF); echo $(SNIFFLES_VCF)) --max_distance 50 --
    max_size_difference -1 --join-mode chr{1..22} | bgzip -c > $(OUTPUT)
tabix $(OUTPUT)
```

Variants that have `NUM_JOINED_SVS` greater than 1 are considered validated SVs. In this analysis we made sure that the type of SV matched between datasets, i.e. the `--ignore-types` option was not used.

syndip comparison We similarly used our merging program, svimmer (`https://github.com/DecodeGenetics/svimmer`), for joining a truth SV set to a query SV callset using the `--join-mode-strict` option.

```
svimmer <(echo $(SYNDIP_VCF); echo $(QUERY_VCF)) --max_distance $(DISTANCE) --
    max_size_difference -1 --join-mode-strict {1..22} > $(OUTPUT_VCF)
```

Only SVs in the syndip high-confidence regions were considered. They were extracted using the `bcftools view` command, using its `--targets-file` option. When extracting SVs out of the query sets, we first extended the high-confidence regions by 25 bp using the `bedtools slop` command, to account for inaccurate SVs.

7 External tools and dependencies

External libraries GraphTyper has the following library dependencies:

- args (`https://github.com/Taywee/args`): Argument parser.
- Boost (`https://www.boost.org/`).
- Catch (`https://github.com/philsquared/Catch`): Framework for unit tests.
- htslib (`https://github.com/samtools/htslib`): Library for HTS data formats.
- paw::Station (`https://github.com/hannespetur/paw`): Multi-threading wrapper library.
- RocksDB (`https://github.com/facebook/rocksdb`): Key-value storage.
- SeqAn[19] (forked version, `https://github.com/hannespetur/seqanhts`): Library for sequence analysis.
- Snappy (`https://github.com/google/snappy`): Compression library.
- SparseHash (`https://github.com/sparsehash/sparsehash`): Hash map containers.
- StatGen (`https://github.com/statgen/libStatGen`): Statistical genetic library.
- zlib (`http://www.zlib.net/`): Compression library.

External programs In addition to the tools we evaluated, we used the following tools in our experiments:

- bamShrink (`https://github.com/DecodeGenetics/bamShrink`): Description below.
- chopBai[20]: Partitions bam index files.
- samtools[14]: Manipulates SAM formatted files.

- vcflib (`https://github.com/vcflib/vcflib`): Manipulates VCF files.
- vt (`http://genome.sph.umich.edu/wiki/Vt`): Manipulates VCF files.

bamShrink bamShrink was run before genotyping with GraphTyper. bamShrink extracts sequence reads of a region and reduces the output file size by binarizing base qualities values, removing unused BAM tags, removing unaligned reads, duplicate reads and reads that have fewer than 40 matching bases in their alignment. In addition to this, bamShrink performs coverage filtering in regions where the coverage is more than 3 times the average coverage, removes Ns if present on either end of a read and removes hard clipped entries from CIGAR strings. Lastly, bamShrink performs adapter removal by clipping overhanging ends of read pairs where the reverse read has been aligned in front of the forward read and their alignments overlap.

Supplementary References

1. Seo, J.-S. *et al.* De novo assembly and phasing of a Korean human genome. *Nature* **538**, 243–247 (2016). URL `http://www.nature.com/doifinder/10.1038/nature20098`.

2. Chaisson, M. J. P. *et al.* Resolving the complexity of the human genome using single-molecule sequencing. *Nature* **517**, 608–611 (2015). URL `http://www.nature.com/articles/nature13907`.

3. Steinberg, K. M. *et al.* Single haplotype assembly of the human genome from a hydatidiform mole. *Genome Research* **24**, 2066–2076 (2014). URL `http://www.ncbi.nlm.nih.gov/pubmed/25373144http://www.pubmedcentral.nih.gov/articlerender.fcgi?artid=PMC4248323`.

4. Evaluation of GRCh38 and de novo haploid genome assemblies demonstrates the enduring quality of the reference assembly. *Genome research* **27**, 849–864 (2017). URL `http://www.ncbi.nlm.nih.gov/pubmed/28396521http://www.pubmedcentral.nih.gov/articlerender.fcgi?artid=PMC5411779`.

5. Shi, L. *et al.* Long-read sequencing and de novo assembly of a Chinese genome. *Nature Communications* **7**, 12065 (2016). URL `http://www.nature.com/articles/ncomms12065`.

6. Nanopore sequencing and assembly of a human genome with ultra-long reads. *Nature Biotechnology* **36**, 338–345 (2018). URL `http://www.nature.com/articles/nbt.4060`.

7. Steinberg, K. M. *et al.* High-Quality Assembly of an Individual of Yoruban Descent. *bioRxiv* 067447 (2016). URL https://www.biorxiv.org/content/10.1101/067447v1.abstract.

8. Eggertsson, H. *et al.* Graphtyper enables population-scale genotyping using pangenome graphs. *Nature Genetics* **49** (2017).

9. McKenna, A. *et al.* The Genome Analysis Toolkit: A MapReduce framework for analyzing next-generation DNA sequencing data. *Genome Research* **20**, 1297–1303 (2010). URL http://genome.cshlp.org/cgi/doi/10.1101/gr.107524.110. arXiv:1011.1669v3.

10. Li, H. & Durbin, R. Fast and accurate short read alignment with burrows–wheeler transform. *Bioinformatics* **25**, 1754–1760 (2009).

11. Eberle, M. A. *et al.* A reference data set of 5.4 million phased human variants validated by genetic inheritance from sequencing a three-generation 17-member pedigree. *Genome Res.* **27**, 157–164 (2017).

12. Chen, X. *et al.* Manta: rapid detection of structural variants and indels for germline and cancer sequencing applications. *Bioinformatics* **32**, 1220–1222 (2015).

13. Parikh, H. *et al.* svclassify: a method to establish benchmark structural variant calls. *BMC Genomics* **17**, 64 (2016).

14. Li, H. *et al.* The sequence alignment/map format and SAMtools. *Bioinformatics* **25(16)**, 2078–2079 (2009).

15. Rausch, T. *et al.* Delly: structural variant discovery by integrated paired-end and split-read analysis. *Bioinformatics* **28**, i333–i339 (2012).

16. Sibbesen, J. A., Maretty, L., Consortium, T. D. P.-G. & Krogh, A. Accurate genotyping across variant classes and lengths using variant graphs. *Nature Genetics* **50**, 1054–1059 (2018).

17. Layer, R. M., Chiang, C., Quinlan, A. R. & Hall, I. M. Lumpy: a probabilistic framework for structural variant discovery. *Genome Biol.* **15**, R84 (2014).

18. Chiang, C. *et al.* Speedseq: ultra-fast personal genome analysis and interpretation. *Nature Methods* **12**, 966–968 (2015).

19. Döring, A., Weese, D., Rausch, T. & Reinert, K. SeqAn an efficient, generic C++ library for sequence analysis. *BMC bioinformatics* **9**, 11 (2008).

20. Kehr, B. & Melsted, P. chopBAI: BAM index reduction solves I/O bottlenecks in the joint analysis of large sequencing cohorts. *Bioinformatics* **32**, 2202–2204 (2016).
