# Supplementary material for: GraphTyper2 enables population-scale genotyping of structural variation using pangenome graphs
Source: Nat Commun. 2019 Nov 27;10:5402. doi: 10.1038/s41467-019-13341-9 (PMC6881350; doi:10.1038/s41467-019-13341-9)
Supplement: Supplementary file 3 — Reporting Summary [file 41467_2019_13341_MOESM3_ESM.pdf]

nature research

Corresponding author(s): Hannes P Eggertsson

Last updated by author(s): Oct 12, 2019

Reporting Summary

Nature Research wishes to improve the reproducibility of the work that we publish. This form provides structure for consistency and transparency in reporting. For further information on Nature Research policies, see Authors & Referees and the Editorial Policy Checklist .

Statistics

For all statistical analyses, confirm that the following items are present in the figure legend, table legend, main text, or Methods section.

n/a	Confirmed	
[ ]	[x]	The exact sample size (*n*) for each experimental group/condition, given as a discrete number and unit of measurement
[x]	[ ]	A statement on whether measurements were taken from distinct samples or whether the same sample was measured repeatedly
[x]	[ ]	The statistical test(s) used AND whether they are one- or two-sided<br>*Only common tests should be described solely by name; describe more complex techniques in the Methods section.*
[x]	[ ]	A description of all covariates tested
[x]	[ ]	A description of any assumptions or corrections, such as tests of normality and adjustment for multiple comparisons
[x]	[ ]	A full description of the statistical parameters including central tendency (e.g. means) or other basic estimates (e.g. regression coefficient) AND variation (e.g. standard deviation) or associated estimates of uncertainty (e.g. confidence intervals)
[x]	[ ]	For null hypothesis testing, the test statistic (e.g. *F*, *t*, *r*) with confidence intervals, effect sizes, degrees of freedom and *P* value noted<br>*Give P values as exact values whenever suitable.*
[x]	[ ]	For Bayesian analysis, information on the choice of priors and Markov chain Monte Carlo settings
[x]	[ ]	For hierarchical and complex designs, identification of the appropriate level for tests and full reporting of outcomes
[x]	[ ]	Estimates of effect sizes (e.g. Cohen's *d*, Pearson's *r*), indicating how they were calculated

Our web collection on statistics for biologists contains articles on many of the points above.

Software and code

Policy information about availability of computer code

Data collection: The Icelandic samples were whole-genome sequenced at deCODE Genetics using Illumina GAIIx, HiSeq, HiSeqX and NovaSeq sequencing machines, and sequences were aligned to the human reference genome (GRCh38) using BWA-MEM (v0.7.10).

Data analysis: GraphTyper is available at: https://github.com/DecodeGenetics/graphtyper (GNU GPLv3 license). The SV merging software is available at: https://github.com/DecodeGenetics/svimmer (GNU GPLv3 license).

For manuscripts utilizing custom algorithms or software that are central to the research but not yet described in published literature, software must be made available to editors/reviewers. We strongly encourage code deposition in a community repository (e.g. GitHub). See the Nature Research guidelines for submitting code & software for further information.

Data

Policy information about availability of data

All manuscripts must include a data availability statement. This statement should provide the following information, where applicable:

- Accession codes, unique identifiers, or web links for publicly available datasets
- A list of figures that have associated raw data
- A description of any restrictions on data availability

Access to the raw Icelandic sequence data, that support the findings of this study, is available on request from KS. The data are not publicly available because of Icelandic state law. Illumina reads for the synthetic-diploid CHM1/CHM13 sample is in the European Nucleotide Archive under accession PRJEB13208. The syndip dataset was obtained from https://github.com/lh3/CHM-eval. The short-read sequences for NA12878, NA12891 and NA12892 were obtained from the Platinum Genome project and the deletion truth set for NA12878 was obtained from the Supplementary Information of svclassify's article.

Field-specific reporting

Please select the one below that is the best fit for your research. If you are not sure, read the appropriate sections before making your selection.

- [x] Life sciences
- [ ] Behavioural & social sciences
- [ ] Ecological, evolutionary & environmental sciences

For a reference copy of the document with all sections, see nature.com/documents/nr-reporting-summary-flat.pdf

Life sciences study design

All studies must disclose on these points even when the disclosure is negative.

Sample size	The samples size used in the study were not predetermined.
Data exclusions	No data were excluded from the analyses.
Replication	The findings can be replicated by using the same computer code. The source code is available on Github (see above).
Randomization	Not relevant, we did not group allocate the participants in the study.
Blinding	The investigators did not take any part in selecting participants.

Reporting for specific materials, systems and methods

We require information from authors about some types of materials, experimental systems and methods used in many studies. Here, indicate whether each material, system or method listed is relevant to your study. If you are not sure if a list item applies to your research, read the appropriate section before selecting a response.

Materials & experimental systems

n/a	Involved in the study	
x		Antibodies
x		Eukaryotic cell lines
x		Palaeontology
x		Animals and other organisms
	x	Human research participants
x		Clinical data

Methods

n/a	Involved in the study	
x		ChIP-seq
x		Flow cytometry
x		MRI-based neuroimaging

Human research participants

Policy information about studies involving human research participants

Population characteristics	There are no covariate-relevant characteristics of the human research participants.
Recruitment	The Icelandic participants were recruited by deCODE genetics. We do not believe there are any recruitment biases that are likely to impact the results of the study.
Ethics oversight	All participating individuals, or their guardians, gave their informed consent before samples were drawn. All sample identifiers were encrypted in accordance with the regulations of the Icelandic Data Protection Authority. Approval for these studies was provided by the National Bioethics Committee and the Icelandic Data Protection Authority.

Note that full information on the approval of the study protocol must also be provided in the manuscript.
